# The structure of the nucleoprotein of Influenza D shows that all *Orthomyxoviridae* nucleoproteins have a similar NP_CORE_, with or without a NP_TAIL_ for nuclear transport

**DOI:** 10.1038/s41598-018-37306-y

**Published:** 2019-01-24

**Authors:** Amélie Donchet, Justine Oliva, Alice Labaronne, Laura Tengo, Myriam Miloudi, Francine C.A. Gerard, Caroline Mas, Guy Schoehn, Rob W.H. Ruigrok, Mariette Ducatez, Thibaut Crépin

**Affiliations:** 1 Institut de Biologie Structurale (IBS), Univ. Grenoble Alpes, CEA, CNRS, 38044 Grenoble, France; 2 IHAP, Université de Toulouse, INRA, ENVT, Toulouse, France; 3 Integrated Structural Biology Grenoble (ISBG) - UMS 3518 (CNRS-CEA-UJF-EMBL), 38044 Grenoble, France

## Abstract

This paper focuses on the nucleoprotein (NP) of the newly identified member of the *Orthomyxoviridae* family, Influenza D virus. To date several X-ray structures of NP of Influenza A (A/NP) and B (B/NP) viruses and of infectious salmon anemia (ISA/NP) virus have been solved. Here we purified, characterized and solved the X-ray structure of the tetrameric D/NP at 2.4 Å resolution. The crystal structure of its core is similar to NP of other Influenza viruses. However, unlike A/NP and B/NP which possess a flexible amino-terminal tail containing nuclear localization signals (NLS) for their nuclear import, D/NP possesses a carboxy-terminal tail (D/NP_TAIL_). We show that D/NP_TAIL_ harbors a bipartite NLS and designed C-terminal truncated mutants to demonstrate the role of D/NP_TAIL_ for nuclear transport.

## Introduction

In 2011, a virus was isolated from a pig with Influenza-like symptoms in Oklahoma (USA). Electron microscopy showed features of an Influenza virus particle and real-time RT-PCR revealed that it was neither an Influenza A virus (IAV) nor an Influenza B virus (IBV). Next-generation sequencing analyses allowed the identification of RNA segments with around 50% identity to human Influenza C virus (ICV). Further serological analyses showed that antibodies against this new virus failed to cross-react with IAV, IBV or ICV (1). All these results suggested it was a new genus of the *Orthomyxoviridae*, temporarily named C/swine/Oklahoma/1334/2011 (C/swine/OK) and then Influenza D virus (IDV). IDV is widely distributed around the world; it was detected in North America^1–4^, Asia^5–7^, Europe^8–10^ and Africa^11^. A serological study in Nebraska (USA) found a seroprevalence of 80% in cattle serum from 2003^3^.

The *Orthomyxoviridae* family includes different *genera*, Influenza A, B, C and D viruses, infectious salmon anemia (ISA) virus, Thogoto virus, Quaranjavirus and others. These viruses are segmented negative strand RNA viruses. Their genomes are made of a set of RNA segments coated with multiple copies of the nucleoprotein (NP) and associated to the viral heterotrimeric polymerase. The number of vRNA segments is specific to each type of Influenza viruses and related to the number of glycoproteins at the surface of the viral particle, 8 segments for IAV and IBV, 7 for IVC and IVD and 6 for Thogoto virus. These ribonucleoproteins (RNPs) are competent for both transcription and replication. To date, several X-ray structures of NP have been published, each of them without RNA. There are three structures of Influenza A nucleoprotein (A/NP): two with NP assembled as a trimer^12,13^ and one for the monomeric R416A mutant^14^. The X-ray structure of the tetrameric Influenza B nucleoprotein (B/NP) is also known^15^, whereas the structure of Isavirus NP (ISA/NP) was solved as a dimer^16^. The overall folds of A/NP and B/NP are very similar with a root-mean-square deviation (rmsd) of 1.6 Å.

Influenza viruses transcribe and replicate in the nucleus of the infected cells and the NPs and the polymerase subunits need to interact with the nuclear transport system of the cell. For Influenza A and B viruses, several studies have shown that the nuclear localization signals (NLS) of NP recognized by the cellular importins-α, are located within the flexible N-terminal tail^17–24^. Recently, crystal structures of the two NLSs of A/NP bound to importin-α have been solved^25,26^.

In this paper, we characterized D/NP and solved the X-ray structure of its tetramer. The C-terminal D/NP_TAIL_ harbouring a classical bipartite nuclear localization signal (NLS) was not visible in the structure. We designed two C-terminal truncated mutants (D/NP-511 and D/NP-529) to study the interaction of D/NP with importin-α7. Our biochemical experiments demonstrate that D/NP_TAIL_ is involved in the interaction with importins-α and immunofluorescence showed that the wild-type D/NP goes into the nucleus whereas the mutants stay in the cytoplasm.

## Results

### Recombinant D/NP forms a tetramer in solution

**Figure 1:**
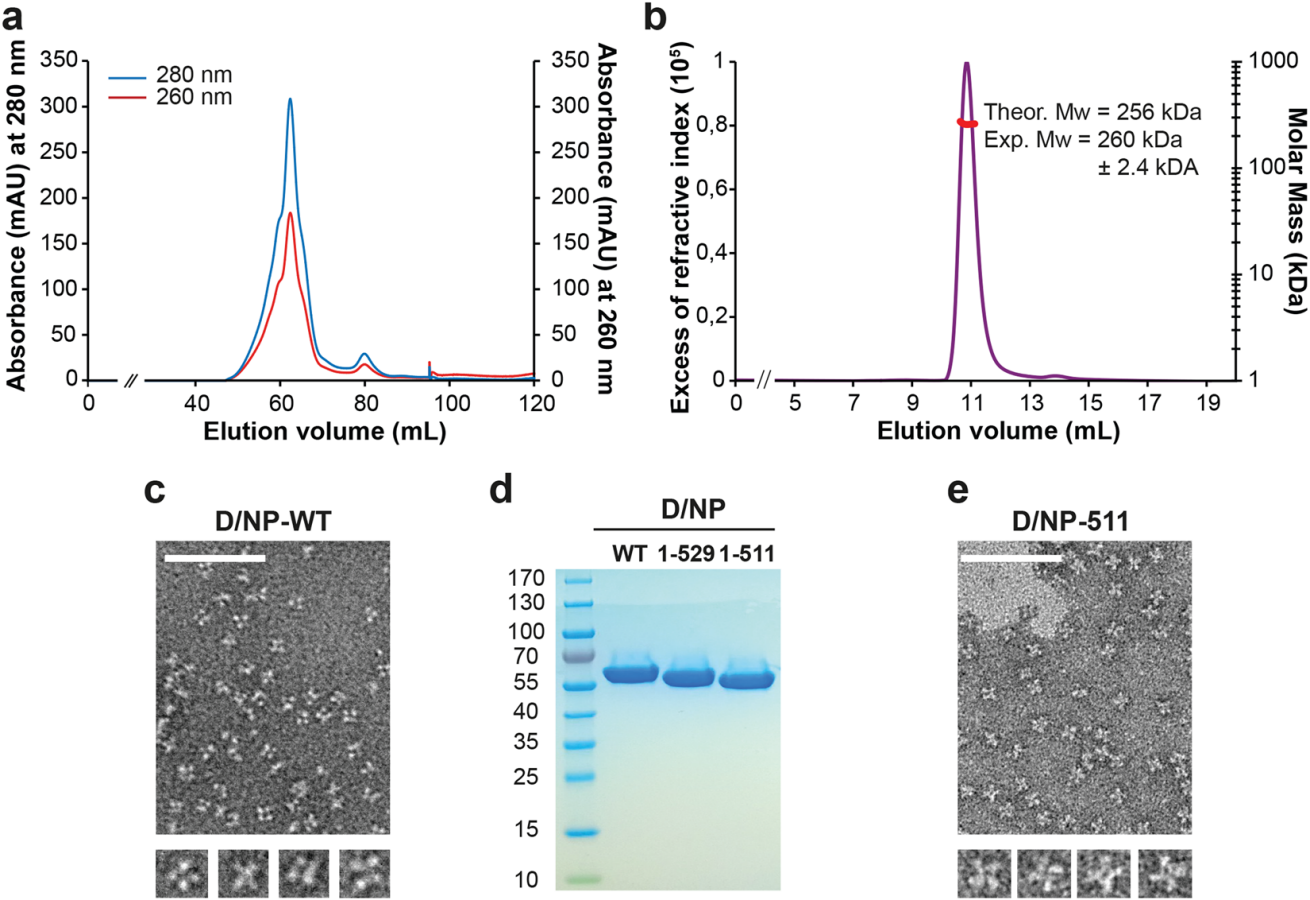
Purification and characterized of Influenza D nucleoprotein. (**a**) Size exclusion chromatography profile of wild-type D/NP. The sample was loaded on a Hiload^TM^ 16/600 S200 column equilibrated with the running buffer 20 mM Tris-HCl pH 7.5, 300 mM NaCl and 5 mM β-mercaptoethanol. (**b**) SEC-MALLS-RI analysis of D/NP. SEC was performed with a Superdex^TM^ 200 increase 10/300 GL column equilibrated with 20 mM Tris-HCl pH 7.5, 150 mM NaCl and 5 mM β-ME. The panel shows the theoretical Mw and the measured Mw. (**c**) and (**e**) Electron microscopy images of the elution peak of D/NP and D/NP-511. Samples show different oligomeric states although most oligomers are tetramers. The scale bar corresponds to 100 nm. (**d**) Coomassie blue-stained SDS-PAGE (4–20% gradient polyacrylamide) showing the purified wild-type D/NP and the two C-terminal truncated mutants (D/NP-529 and D/NP-511).

The DNA coding sequence of D/NP was cloned in a bacterial expression plasmid and over-expressed in *Escherichia coli* as a C-terminal His-tagged recombinant protein. D/NP was purified with a nickel affinity chromatography followed by a heparin column and a final gel filtration. Figure 1a shows a typical gel filtration elution profile using the absorbance signals at 260 and 280 nm (ratio 280/260 > 1.75). The protein could then be concentrated at 2 to 6 mg.mL^−1^. Polyacrylamide gels and SEC-MALLS-RI experiments have confirmed the homogeneity and the molecular weight of the recombinant tetrameric D/NP (Fig. 1b,d). By electron microscopy (negative staining), we showed that D/NP forms mainly tetramers in solution (Fig. 1c). Previously, it was shown that the oligomerization of recombinant A/NP and B/NP can be modulated by the NaCl concentration^14,18,27,28^. Starting from purified and stable oligomeric samples (trimers for A/NP and tetramers for B/NP), monomeric proteins can be obtained by decreasing stepwise the NaCl concentration. After purification at 300 mM NaCl followed by dialysis at 150 mM NaCl, D/NP was eluted from gel filtration (with a 150 mM NaCl running buffer) in the same volume, meaning that a smooth reduction of the salt concentration did not change its oligomeric state. However, a decrease to 50 mM NaCl induced an irreversible and total precipitation of D/NP, even with a stepwise reduction at 150 mM NaCl. Therefore, the experiments on D/NP and its mutants were carried out at 300 mM NaCl.

Like the nucleoproteins of A, B and ISA, D/NP bound single-stranded RNA. We measured the *Kd* of 14 nM with a fluorescence anisotropy assay with an RNA of 24 nucleotides of polyUC labeled in 3’ with 6-fluorescein amidite (FAM)^27^. The *Kd* of 14 nM can be compared with the Kds of 7 nM of the trimer of A/NP, 31 nM of the tetramer of B/NP^29^ and 24 nM for the dimer of ISA/NP^16^.

### Structure of D/NP

**Figure 2:**
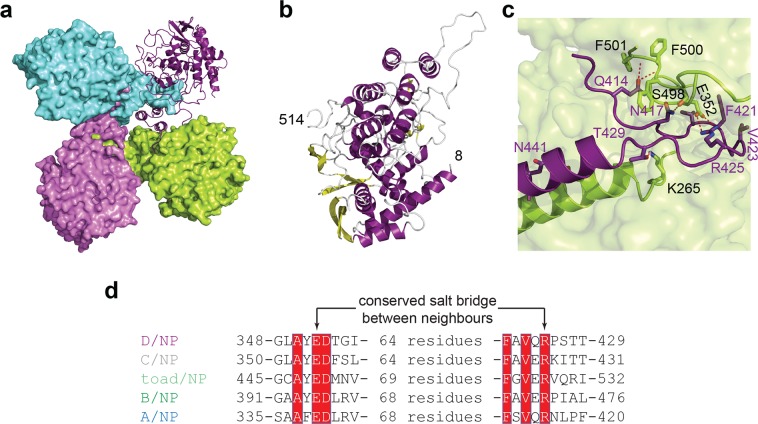
Structure of Influenza D nucleoprotein. (**a**) Structure of the tetrameric D/NP, with 3 protomers shown in surface (respectively in green, pink and cyan) and the fourth in cartoon (deep purple). (**b**) Cartoon representation of one monomer of D/NP with the α-helixes in deep purple and β-strands in yellow. (**c**) Detail of the interactions between two protomers of the tetrameric D/NP as shown in (**a**). The conserved R425 of one protomer (shown as deep purple cartoon) stabilized the position of the oligomerization loop at the surface of the neighbouring protomer, through its conserved E352. (**d**) Sequence alignment of the salt bridges of the oligomerization-loop of one protomer to the NP_CORE_ of the neighbour protomers. For the sequences see Table 1.

Full length D/NP was crystallized in sodium malonate as small fine needles that diffract X-rays up to 2.4 Å resolution (Supplementary Table 1). The structure was solved by molecular replacement using a starting model of the monomer of the A/NP R416A mutant^14^. The automatic search gave the position for three molecules and a fourth protomer was fitted manually after the analysis of the electron density. The molecular replacement using the tetrameric B/NP model did not give any acceptable solutions. The asymmetric unit contained four molecules arranged as a tetramer (Fig. 2a and refinement statistics in Supplementary Table 1), consistent with the observations made by electron microscopy. Based on the electron density, 87% of the model could be built, from residues 8 to 514 (Fig. 2b). Several internal loops were missing and the C-terminal 50 residues were disordered. The oligomerization-loop of one protomer plugged into a cavity of its neighbour (Fig. 2c). The N-terminal part of the loop started with a strictly conserved glutamate residue (Gln-414) that interacted with the backbone of the strictly conserved consecutive aromatic residues _499_FFF_501_. The loop was then stabilized by several kind of contacts; mainly hydrophobic (by the side chain of the strictly conserved Phe-421 and Val-423) and of several aromatic residues lying in the pocket of the neighbouring monomer but also the salt bridge between an arginine from one monomer (Arg-425) interacting with a glutamate of the neighbour (Glu-352; Fig. 2c,d). These two residues, also strictly conserved in all Influenza NPs (Fig. 2d and Supplementary Fig. 1), are well known for modulating their oligomeric state. Once mutated in alanine, the corresponding single mutants formed monomers, unable to self-assemble^13,14,28,30–33^. Phosphorylation state of A/NP has been shown to also be a key factor of NP/NP interfaces, especially for positions Ser-165 and Ser-407^34,35^. Sequence alignment showed that D/NP contains a threonine (Thr-161) and a serine (Ser-416) respectively, suggesting a conserved mechanism for the regulation of the oligomerization (Supplementary Fig. 1).

### Core structure of all nucleoproteins (NP_CORE_) with tails (NP_TAIL_)

**Figure 3:**
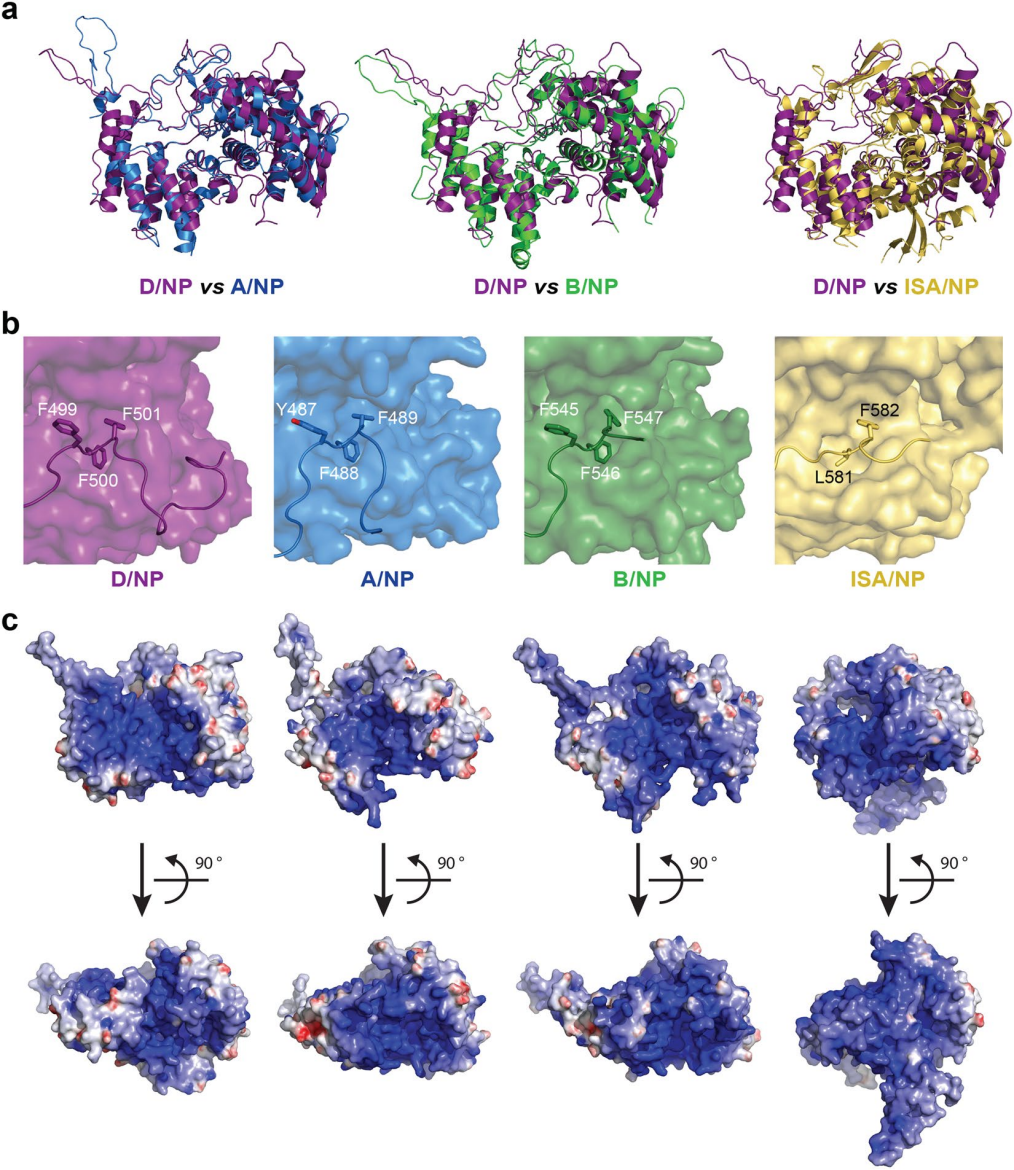
Comparison of D/NP with other segmented negative sRNA virus nucleoproteins. (**a**) The structure of one protomer of D/NP (deep purple) has been superimposed from left to right, with one protomer of A/NP (blue; PDB id: 2IQH), B/NP (forest; PDB id: 3TJ0) and ISA/NP (light orange; PDB id: 4EWC). The rmsd values are given in Table 1. (**b**) Anchoring of the C-terminus on NP_CORE_ by a patch of 3 consecutive aromatic residues. The panel corresponds to a zoom of the superimposed structures shown in panel A with NP_CORE_ represented in surface. (**c**) Electrostatic surface potentials of one protomer of D/NP, A/NP, B/NP and ISA/NP. The electrostatic surfaces were calculated from the crystal structures using DelPhi^75^. The potential scales range from −10.0 kT/e (red) to 10.0 kT/e (blue).

The overall folds of A/NP, B/NP, ISA/NP and D/NP were very similar (Fig. 3a) with root-mean-square deviations (rmsd) between 1.6 Å (for 383 Cα for the comparison A/NP with B/NP) and 2.9 Å (for 340 Cα for the comparison D/NP with ISA/NP, Table 1). Including ISA/NP, a common architecture for all Influenza-like NPs can be defined based on the X-ray structures with the NP_CORE_ starting with the first α-helix for A/NP, B/NP and D/NP and finishing with three hydrophobic residues, anchored into the surface of the protein (Supplementary Fig. 1). Figure 3b shows the X-ray structures of A/NP, B/NP, D/NP and ISA/NP with a zoom on the C-terminal regions, where a superimposed patch made by three consecutive aromatic residues is found in Influenza NPs (_487_YFF_489_ for A/NP, _545_FFF_547_ for B/NP and _499_FFF_501_ for D/NP). A similar patch is also present in the ISA/NP model (_580_GLF_582_). The NP_CORE_ contains the large and shallow positively charged surface which might bind RNA (Fig. 3c), without sequence specificity (see above)^12,13,36,37^. Twenty or 71 residues without any structure are found N-terminally before the cores of A/ and B/NP whereas for D/NP, the core starts with a very short version of an N-terminal tail (only 7 residues), which seems to be compensated by the presence of a carboxy-terminal NP_TAIL_ of 51 residues (Supplementary Fig. 1). The length of the NP_TAILs_ is variable and its location seems to be specific for each genus. Because D/NP differs from A/ and B/NP in its global organization, we decided to further analyse the C-terminal D/NP_TAIL_.

**Table 1:** Nucleoproteins of Orthomyxoviruses.

	A/NP	B/NP	toad/NP	C/NP	D/NP	ISA/NP
A/NP		1.6 Å (383 Cα)	—	—	2.1 Å (356 Cα)	2.9 Å (340 Cα)
B/NP	38%		—	—	1.9 Å (365 Cα)	2.8 Å (340 Cα)
toad/NP	25%	30%			—	—
C/NP	22%	25%	24%		—	—
D/NP	24%	25%	23%	38%		2.9 Å (330 Cα)
ISA/NP	20%	18%	18%	16%	20%	
Tho/NP	18%	21%	20%	18%	17%	20%
WfB/NP	20%	19%	19%	20%	18%	17%

A/NP, Influenza A virus nucleoprotein, strain A/WSN/1933(H1N1) (Uniprot accession number B4URF1, PDB entry 2IQH); B/NP, Influenza B virus nucleoprotein, strain B/Managua/4577.01/2008 (Uniprot accession number C4LQ26, PDB entry 3TJ0); Toad/NP, Wuhan asiatic toad Influenza virus nucleoprotein (GenBank accession number AVM87634); C/NP, Influenza C virus nucleoprotein, strain C/AnnArbor/1/1950 (Uniprot accession number Q6I7C0); D/NP, Influenza D virus nucleoprotein, strain D/bovine/France/2986/2012 (Uniprot accession number A0A0E3VZU8, PDB entry 5N2U); ISA/NP, infectious salmon anemia virus nucleoprotein, isolate salmon/Norway/810/9/99 (Uniprot accession number Q8V3T7, PDB entry 4EWC); Tho/NP, Thogoto virus nucleoprotein (Uniprot accession number A0A0B6VKB5); WfB/NP, Wellfleet Bay virus nucleoprotein (Uniprot accession number A0A0A1E9N5). Sequence identities were obtained using MUSCLE^77^ and structure comparisons were calculated using PDBeFold^78^.

### D/NP_TAIL_ behaves as an intrinsically disordered protein

**Figure 4:**
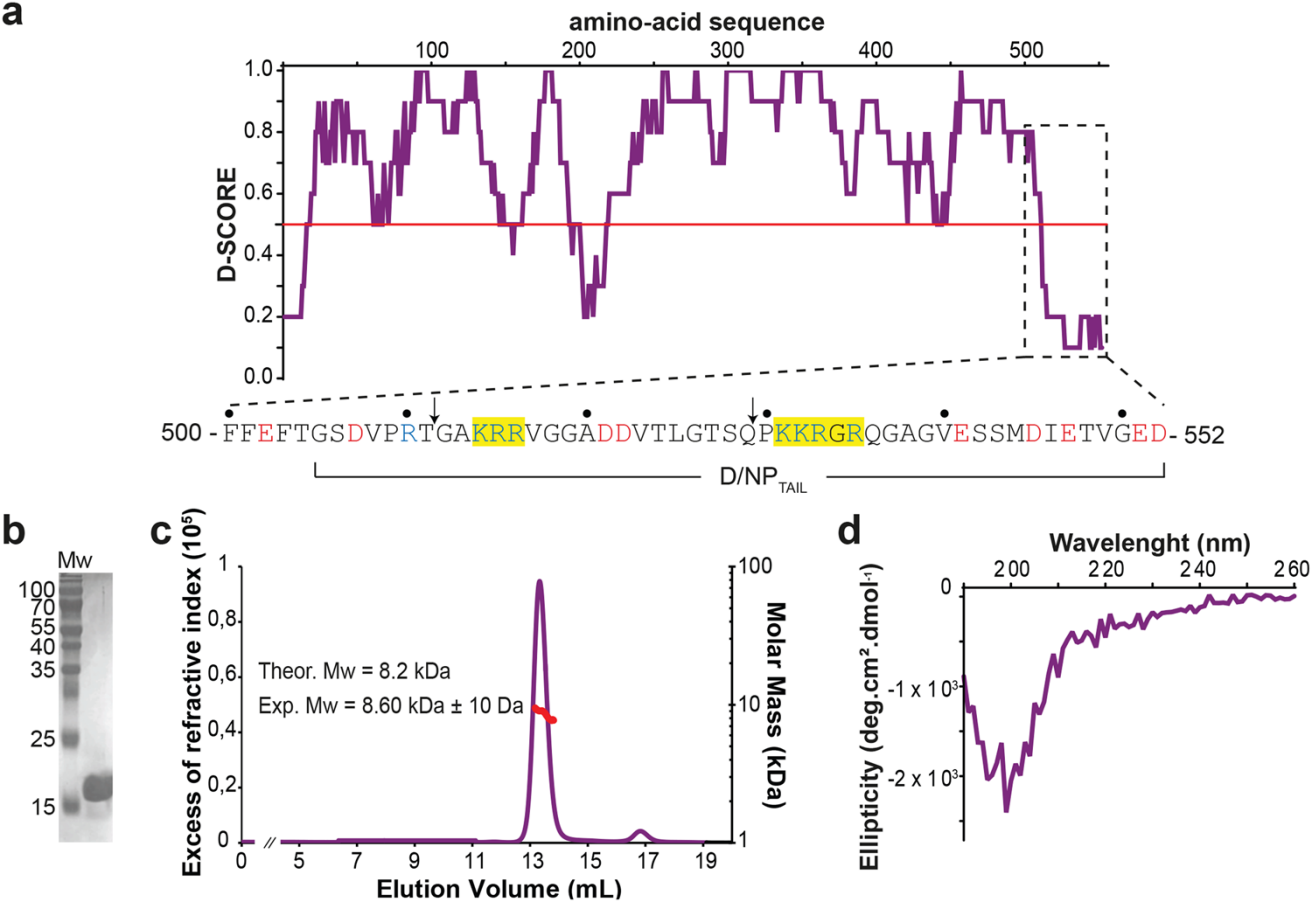
Biophysical characterization of D/NP_TAIL_. (**a**) D-score (score for disorder as a function of residue) of D/NP with a zoom (below the graph) on the last 50 residues. The prediction is based on 22 predictor web servers and the D-score was calculated by adding the values for each residue and dividing by the number of used algorithms. We arbitrarily defined a threshold level at 0.50; residues with a D-score <0.50 were assigned as disordered^38^. The yellow boxes on the sequence are to highlight the putative NLS motifs. The arrows indicate where the sequences were cut for making D/NP-529 and D/NP-511. (**b**) Coomassie blue-stained SDS-PAGE (Tris-Tricine; 15% polyacrylamide) of the purified D/NP_TAIL_. It migrates at a higher molecular weight (17 kDa approximately) than expected (8 kDa). (**c**) SEC-MALLS-RI analysis of D/NP_TAIL_ loaded on S75 10/300 GL column. For this experiment, we have chosen to keep the His-tag encoded with the pETM11 plasmid, for an optimal detection of D/NP_TAIL_ with UV. The experimental molecular weight is consistent with the expected mass. (**d**) Circular dichroïsm of D/NP_TAIL_. CD is a biophysical method based on the polarization of light, used for a fast determination of the secondary structures within the proteins in solution. α-Helical proteins show negative bands at 222 nm and 208 nm and a positive band at 193 nm, proteins with well-defined antiparallel β-sheets have negative bands at 218 nm and positive bands at 195 nm and disordered proteins have very low ellipticity above 210 nm and negative bands near 195 nm.

Based on the crystal structure and a disorder prediction (Fig. 4a), we designed a construct (from residues 505 to 552) for the expression in *E*. *coli* as an N-terminal His-tagged recombinant version. Considering that the core of D/NP ends after residue Phe-501, we have chosen to start the construct at Gly-505, in order to avoid the hydrophobicity of Phe-503 (Fig. 4a,b). D/NP_TAIL_ was eluted from gel filtration as a 40 kDa-protein (data not shown) but with a normal migration pattern on SDS-PAGE (Fig. 4b). As intrinsically disordered proteins are known to be eluted from gel filtration with an aberrant volume^38,39^, a SEC-MALLS-RI experiment (Fig. 4c) confirmed the monodispersity of the purified sample with a Mw of 8.2 kDa for D/NP_TAIL_ (with the His-tag). A circular dichroism analysis confirmed that D/NP_TAIL_ did not contain any significant stable secondary structure in solution (Fig. 4d), even in presence of TMAO (Supplementary Fig. 2), a chemical known to force unfolded proteins to fold to native-like species with significant functional activity^40–42^.

### D/NP_TAIL_ interacts with importin-α

**Figure 5:**
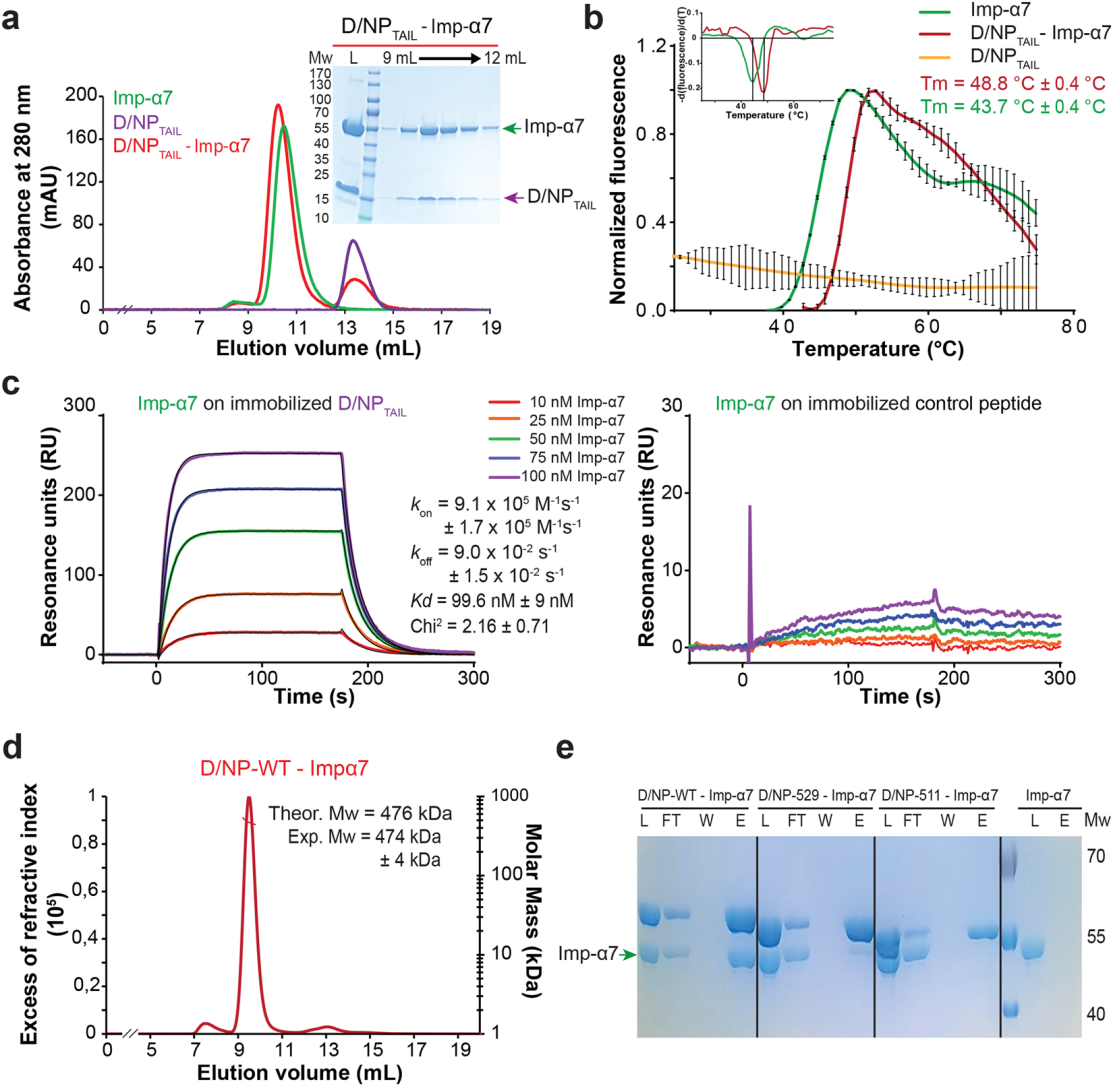
Interaction of D/NP and D/NP_TAIL_ with importin-α7. (**a**) Size exclusion chromatography profile of a mixture between human importin-α7 and D/NP_TAIL_. The mixture (molar ratio 1 importin-α7 for 2 D/NP_TAIL_) was incubated 1 hour at room temperature and then loaded on a Superdex^TM^75 10/300GL column equilibrated with the running buffer 20 mM Tris-HCl pH 7.5, 250 mM NaCl, 5 mM β-mercaptoethanol. (**b**) Thermal stability assay of importin-α7 in absence (green) or in presence (red) of D/NP_TAIL_ using Thermofluor^76^. In presence of D/NP_TAIL_, the melting temperature of importin-α7 is 5 °C higher. D/NP_TAIL_ alone using Thermofluor did not give any denaturation signal (yellow curve). The upper insert corresponds to the derivative of the fluorescence signal for a precise measure of the melting temperature. (**c**) Affinity of importin-α7 for D/NP_TAIL_ by measured by surface plasmon resonance (SPR). Biotinylated D/NP_TAIL_ (left) and control peptide (right) were captured on a streptavidin-coated sensor chip surface before injections of several importin-α7 concentrations (10 nM in red, 25 nM in orange, 50 nM in green, 75 nM in blue and 100 nM in purple). The sensorgrams of the interaction between D/NP_TAIL_ and importin-α7 were fitted under a Langmuir 1:1 binding model with mass-transfer (black line). (**d**) SEC-MALLS analysis of D/NP in complex with importin-α7. The mixture (molar ratio 1 D/NP for 1.2 importin-α7) was incubated 1 hour at room temperature and then loaded on a Superdex^TM^ 200 increase 10/300 GL. The experimental molecular weight is consistent with the expected mass of four importins-α7 bound per D/NP tetramer. (**e**) Pull-down assays of human importin-α7 by D/NP and the two C-terminal truncated mutants (D/NP-529 and D/NP-511). The his-tags are on D/NP. The mixtures (molar ratio 1 D/NP for 1.2 importin-α7) were incubated 1 hour and the experience was done as described in panel (**a**). The figure shows the coomassie blue-stained SDS-PAGE (12% polyacrylamide) with the Load, FlowThough, Wash and the second fractions (E2).

The N-terminal tails of A/NP and B/NP are known to be involved in the nuclear import by interacting with importins-α^17–19,21,23,25,43^. The sequence analysis of D/NP showed no import signals in its N-terminal part but suggested the presence of a bipartite NLS within D/NP_TAIL_ (_514_KRR-X_14_-KKRGR_535_; Fig. 4a). We thus tested the bipartite NLS with different constructs. First, we showed that the human importin-α7 is co-eluted with the His-tagged D/NP_TAIL_ from gel filtration (Fig. 5a). The interaction was confirmed by thermal-shift experiments, where importin-α7 appeared more stable in presence of D/NP_TAIL_ (Fig. 5b). We also confirmed the interaction between the two partners using the full-length D/NP. Using surface plasmon resonance, we measured a *Kd* of 100 nM between immobilized D/NP_TAIL_ and importin-α7 (Fig. 5c), ten-fold higher that the affinities measured for the N-terminally A/ and B/NP_TAILs_^18,25^. By gel filtration, we showed that the D/NP:importin-α7 complex could be eluted as a single peak. A molecular weight of 474 kDa has been measured by SEC-MALLS-RI for the complex (Fig. 5d), corresponding to the interaction of four molecules of importins-α7 (Mw = 4 × 55 kDa) per tetramer of D/NP (Mw = 260 kDa). To confirm the role of D/NP_TAIL_ in the interaction with importin-α, we designed two C-terminal truncated constructs of D/NP, D/NP-511 and D/NP-529, ending respectively at residues 511 and 529. D/NP-511 is deprived of the whole bipartite NLS whereas D/NP-529 contains only the first part (Fig. 4a). They both behave as the wild-type protein during the purification (Fig. 1d,e). Using pull-down experiments on nickel-affinity resin, we showed that D/NP-529 still slightly retained importin-α7 whereas the interaction between D/NP-511 and importin-α7 was totally abolished (Fig. 5e). This suggests that importin-α7 binds to the NP_TAIL_ of D/NP.

### Nuclear transport of D/NP and its mutants

**Figure 6:**
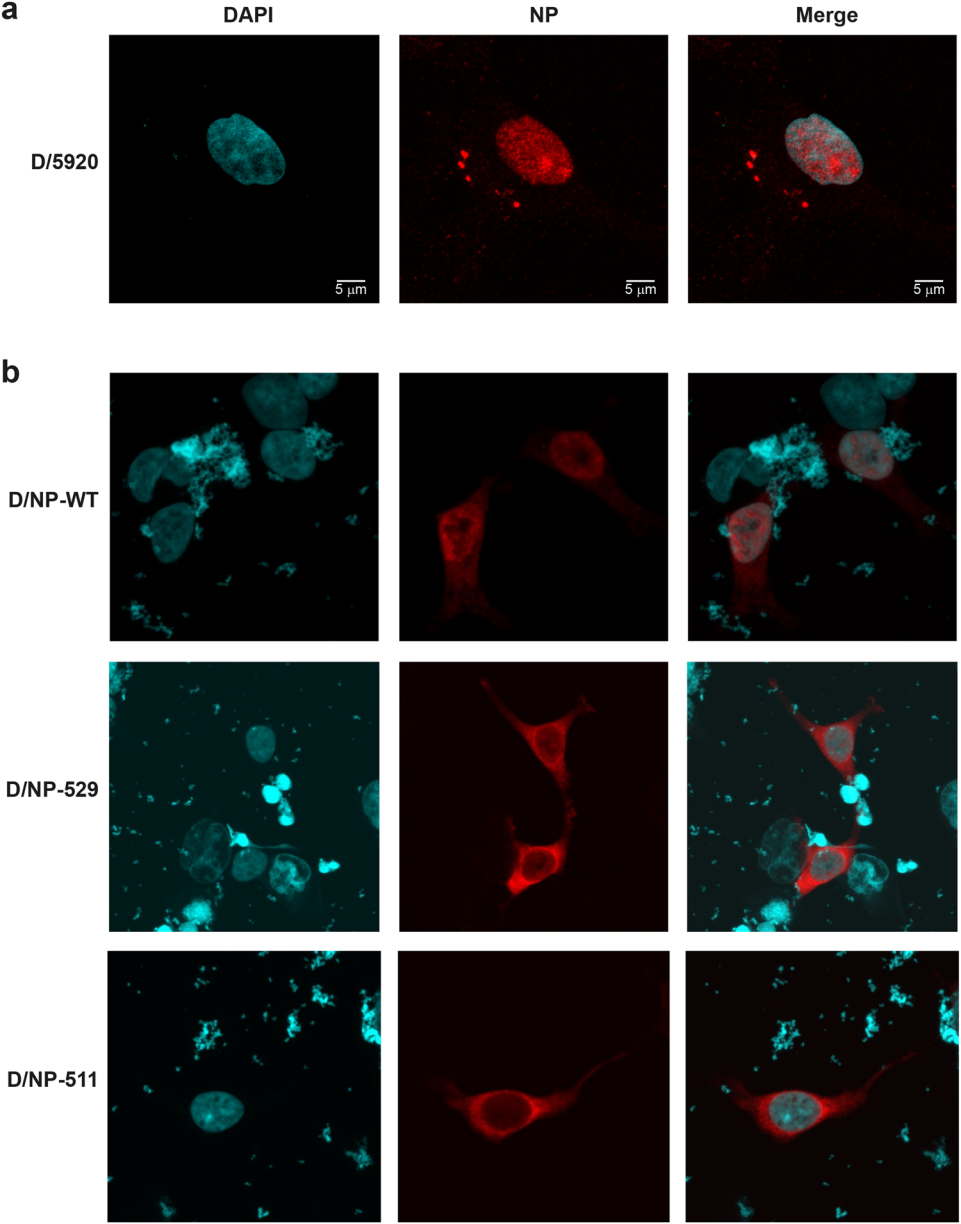
Nuclear transport of D/NP in HEK 293T cells. Microscopy pictures of HEK 293T cells with DAPI, NP and merged DAPI + NP staining (left, middle and right panels, respectively). (**a**) D/NP after infection of HEK 293T cells at 6 hours post infection with a *moi* of 5. (**b**) Cells were transfected with wt D/NP, D/NP-529 and D/NP-511. After 24 h cells were fixed, permeabilized and analyzed by indirect immunofluorescence. NP localization was observed using an in house rabbit hyperimmune NP-IDV serum and anti-rabbit IgG labeled Rhodamine RX (in red). Cells were mounted with DAPI-Vectashield and observed with a Leica Zeiss 710 (magnification: x63). In red: NP protein; in blue: nucleus. The bar in (A) represents 5 µm.

First, human HEK 293T cells were infected with D/bovine/France/5920/2014 (*moi* of 5) and indirect immunofluorescence was used to localize D/NP within the cells. After 6 h, most of D/NP was observed in the nucleus (Fig. 6a). As a control, HEK 293T cells were infected with Influenza A/PR/8/34 and we found very similar results (data not shown). We then transfected HEK 293T cells with a plasmid containing wild-type D/NP and we found that D/NP can be localized in the cytoplasm but mainly in the nucleus (Fig. 6b). With this transfection strategy, D/NP-529 was located mainly in the cytoplasm with a little staining observed in the nucleus, whereas, D/NP-511 was located exclusively in the cytoplasm of the transfected cells (Fig. 6b), clearly showing that the cell used the bipartite NLS between residues 513 and 535 for the nuclear import of D/NP.

## Discussion

In 2011, a new virus has been isolated from pigs exhibiting Influenza-like illness. Subsequent studies identified an *Orthomyxovirus* with seven RNA segments sharing 50% overall amino acids identity with the human Influenza C virus^2^. First considered as a subtype of the Influenza C virus, it has since been officially named Influenza D virus (IDV) by the International Committee of Taxonomy of Viruses in 2016. Since its first isolation, IDV appears to circulate all around the world in many mammals, with cattle as a possible reservoir, and with serological data suggesting it may transmit to humans^44^. The recent structure of IDV Hemagglutinin-Esterase-Fusion glycoprotein (HEF) has described an open receptor-binding cavity capable of accommodating diverse extended glycan moieties that could be one reason for its broad cell tropism^45^.

With this paper, we provide a detailed characterization of IDV nucleoprotein. We determined the structure of the tetramer of D/NP and we failed to find the biochemical conditions to stabilize the monomer. For A and B/NP we had found that the monomer stays in solution at low salt, 50 mM NaCl^14,27,28^ but the D/NP protein makes aggregates in salt conditions lower than 150 mM. Post-translation changes like phosphorylation or ubiquitination may be able to stabilise the monomer, as it has been shown for A/NP^14,34,46,47^. The phosphorylation on A and B/NP are summarized in Supplementary Fig. 1.

**Figure 7:**
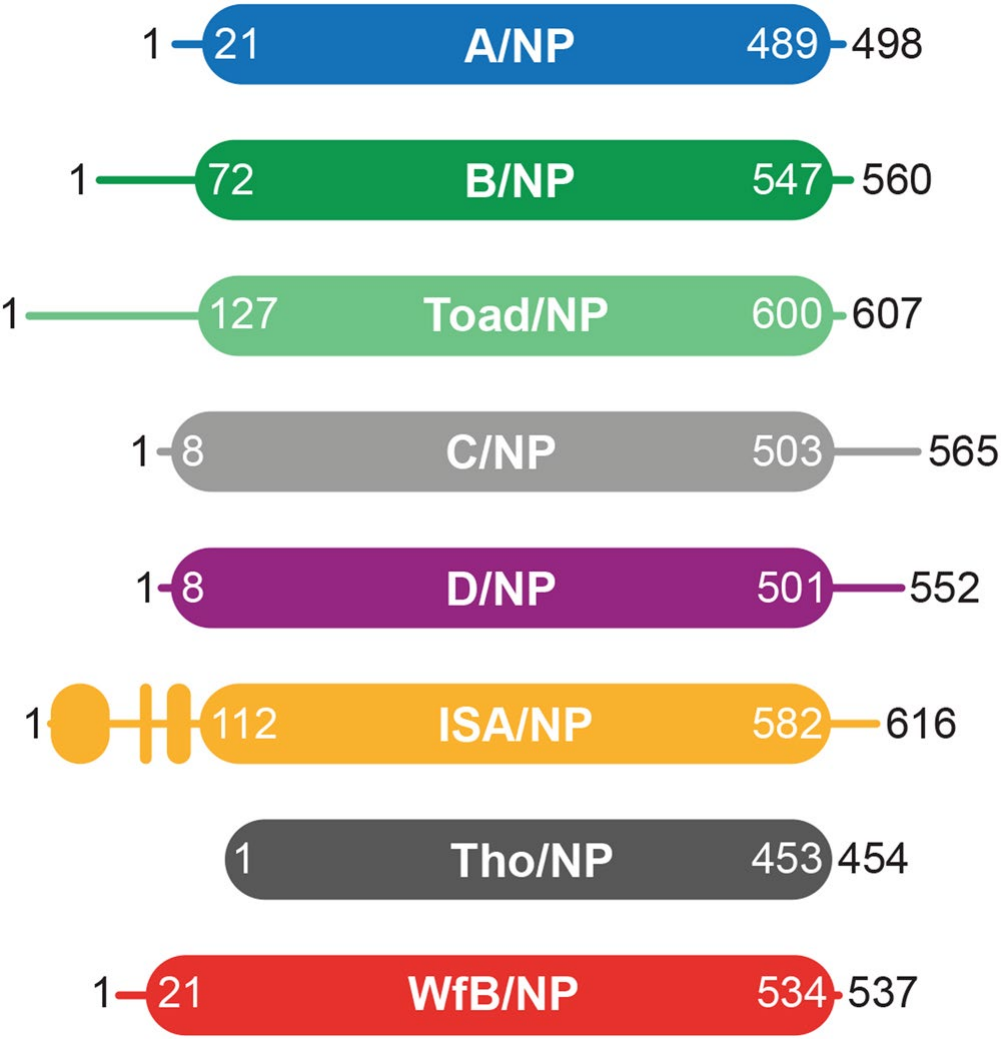
Schemas for nucleoproteins of *Orthomyxoviruses*. Schematic representation of the nucleoprotein based on the amino acid sequence identity and structure analysis of the protein from representative members of the *Orthomyxoviridae* family. The schema respects the size of the proteins. The protein accession numbers are the same as Table 1. The flexible tails are represented with simple lines whereas the folded parts and the cores are represented with filled boxes.

The comparison of the structures of the nucleoproteins of Influenza A, B, D and of infectious salmon anemia virus (ISAV) shows that of all these proteins share a common structural core (NP_CORE_) that start with the first α-helix in the structure of A, B and D, up to three consecutive aromatic residues (Fig. 3 and Supplementary Fig. 1). Looking at the sequences of all known *Orthomyxoviruses* nucleoproteins shows that such hydrophobic patch is present in C/NP (_501_FFF_503_) and could be present in Tho/NP (_451_YLF_453_) and Wfb/NP (Wellfleet bay virus nucleoprotein; _532_VIY_534_). The only structure that differs is the ISAV/NP that has a folded domain upstream of the first α-helix of the NP_CORE_ (Fig. 7). One could also see that Tho/NP is constituted only by the NP_CORE_ without any other appended sequences. NPs of IAV and IBV have an N-terminal NP_TAIL_ whereas the NPs of ICV and IDV have a C-terminal NP_TAIL_. The NP_TAIL_ of A is only 20 residues whereas the tails of the other Influenza viruses NPs are much longer; 71 residues for IBV, 62 for ICV, 51 for IDV. Recently, new large-scale methods for finding RNA viruses in vertebrates other than mammals and birds, identified up to 240 “new” viruses^48^. These authors found new Influenza viruses close to IBV, a Wuhan spiny eel Influenza virus and a Wuhan Asiatic toad Influenza virus. The NP for the toad virus (toad/NP) is very close to B/NP (identity of 30%, Table 1) with a very long N-terminal tail of 126 residues (Fig. 7 and Supplementary Fig. 1). Interestingly, toad/NP possess an alanine residue (Ala-278) aligned with A/NP Ser-165 and D/NP Thr-161 and a phenylalanine (Phe-519) aligned with A/NP Ser-407 and D/NP Ser-416.

We demonstrated that the C-terminal D/NP_TAIL_ presents all the characteristics of an intrinsically disordered protein and that is involved in the nuclear import through its interaction with cellular importins-α. This role is not a big surprise since the presence of such NLS motifs was identified within the flexible NP_TAILs_ of viruses A and B located at the N-terminus of these proteins^17,18,21,23,25,43,49^. Tho/NP has been shown to be imported in the nucleus^50^ but this nucleoprotein does not possess any additional intrinsically disordered protein-like peptide with an NLS. Recently, a second NLS on A/NP (strain A/X-31) at the rim of the RNA binding channel was shown. A crystal structure shows that this internal NLS (_198_Lys-Arg-INDRNFWRGENC-Arg-Arg-T-Arg_216_) binds to mouse importin-α1^26^. Likewise, for the NLSs of Tho/NP and ISA/NP, it has been suggested that the rims on the RNA binding surfaces can be used as an NLS for nuclear import for viral replication^16,50,51^.

Finally, we have shown that the NP_TAILs_ of these nucleoproteins are intrinsically disordered proteins. The preservation of such intrinsically disordered protein-like peptide with a conserved function highlights its importance. The name for N_TAIL_ was first used for the C-terminal disordered tail of measles virus nucleoproteins^52^, which is about 125 residues long. This tail is disordered but can adopt a short α-helical structure (residue D484-A502) to promote the interaction with the phosphoproteins of these viruses which bind themselves to the corresponding RNA polymerase^53,54^. For these viruses, the tails keep the nucleocapsids in a flexible conformation. When cleaved off with trypsin, the helical nucleocapsids become rigid^55,56^. The tails do not change the affinity of the nucleoprotein for RNA but change the kinetics for the assembly of the nucleocapsids^57^. The RNPs of Influenza A and B viruses are also very flexible^58–61^ which makes it difficult to handle for high resolution structures. The EM structure of the measles nucleocapsid with a resolution of 3.6 Å^62^ was performed on the nucleocapsids without the C-terminal N_TAIL_. If we could generate Influenza RNPs with NP without NP_TAIL_ to rigidify the RNPs, it would make it easier solving a high resolution structure to observe the RNA on the nucleoproteins.

## Methods

### Molecular biology for expression of nucleoprotein of Influenza D

The DNA coding sequence of D/bovine/France/2986/2012 NP was bought at GeneArt (ThermoFisher Scientific) optimized for bacterial expression. The different constructs have been PCR amplified and cloned following the supplier procedures (New England Biolabs). D/NP, D/NP-511 and D/NP-529 DNA coding sequences were cloned in pETM13 (EMBL) to express C-terminal His-tagged proteins whereas D/NP_TAIL_ (505-D/NP-552) DNA coding sequence was cloned in pETM11 (EMBL) to express an N-terminal His-tagged fragment. The DNA coding sequence of the human importin-α7 (KPNA6; Uniprot accession number O60684) was cloned without its IBB domain (amino-acids 58 to 536) in pET9a (Novagen) to express an N-terminal His-tagged protein^63^. Sequencing was performed by Eurofins.

### Expression and purification of proteins

*Escherichia coli* BL21 RIL (DE3) cells (Life Technology) were transformed with the resulting plasmids. Cultures were induced 12 h by adding 0.3 mM isopropyl-β-D-thiogalactopyranoside (IPTG) at 18 °C and collected by centrifugation. The pellets were resuspended and sonicated in 50 mM Tris-HCl pH 7.5, 300 mM NaCl, 1 M NDSB-201 (Sigma), 2 mM β-mercaptoethanol (β-ME) and complete protease inhibitor cocktail (Roche) for D/NP constructs and 50 mM Tris-HCl pH 8, 500 mM NaCl, 1 mM β-ME and complete protease inhibitor cocktail for importin-α7. All purifications were performed at room temperature. All D/NP constructs were purified by nickel affinity chromatography (Ni-NTA, Qiagen) followed, in the case of D/NP, D/NP-511 and D/NP-529, by a HiTrap^TM^ Heparin HP column (GE-Healthcare) on a NGC system (BioRad). Elution fractions of D/NP, D/NP-511, D/NP-529 and D/NP_TAIL_ were dialyzed against 20 mM Tris-HCl pH 7.5 at 50 mM or 300 mM NaCl, 5 mM β-ME. The last purification step was a size-exclusion chromatography using a Hiload^TM^ 16/600 S200 column (GE-Healthcare) for D/NP, D/NP-511 and D/NP-529 or a S75 10/300 GL column (GE-Healthcare) for D/NP_TAIL_. Importin-α7 was purified by Nickel affinity chromatography and elution fraction were dialyzed with TEV (1/100) against 20 mM Tris-HCl pH 7.5 at 150 mM NaCl, 5 mM β-ME and 20 mM imidazol. Finally, a size-exclusion chromatography using Superdex^TM^ 200 increase 10/300 GL column (GE-Healthcare) was performed in 20 mM Tris-HCl pH 7.5 at 150 mM NaCl, 5 mM β-ME. Peak fractions were concentrated using a 10 kDa Amicon concentrator. Protein concentrations were determined using the extinction coefficients at 280 nm, ε = 44 537 M^−1^.cm^−1^ for D/NP, ε = 41 558 M^−1^.cm^−1^ for D/NP-529 and D/NP-511, ε = 2980 M^−1^.cm^−1^ for D/NP_TAIL_, and ε = 46 785 M^−1^.cm^−1^ for importin-α7.

### SEC-MALLS-RI analysis

Multi-angle laser light scattering (MALLS) coupled with size exclusion chromatography (SEC) and refractometry (RI) is a method for measuring the absolute molecular mass of a particle in solution that is independent of its dimensions and shape^64^. SEC was performed with a column (Superdex^TM^ 200 increase 10/300 GL or Superdex 75 10/300 GL) equilibrated with 20 mM Tris-HCl pH 7.5, 150 mM NaCl and 5 mM β-ME. Analytical runs were performed at 20 °C with a flow rate of 0.5 mL.min^−1^. MALLS detection was performed with a DAWN-HELEOS II detector (Wyatt Technology) using a laser emitting at 690 nm and protein concentration was measured on-line with the use of differential refractive-index measurements, with an Optilab T-rEX detector (Wyatt Technology) and a refractive-index increment, dn/dc, of 0.185 mL.g^−1^. Weight-average molar masses (Mw) were calculated with ASTRA (Wyatt Technology) as previously described^38^.

### Electron microscopy

Samples (concentrations around 0.05 mg.mL^−1^) were applied between a carbon and a mica layer. The carbon was then floated on the top of a 2% (w/v) sodium silicotungstate, pH 7.0 solution. The carbon film was covered by a copper grid. Both were fished using a small piece of journal paper and air dried before insertion in the electron microscope^65^. Charge-coupled Device (CCD) frames were taken with a FEI T12 microscope operating at 120 kV and a nominal magnification of 45 000 times. The dilutions for EM were performed with the size-exclusion buffer right before preparing the grid.

### Crystallisation and structure determination

D/NP was crystallised by vapor diffusion using the sitting drop method. The crystals were obtained in 2.4 M sodium malonate pH 4 with a protein concentration of 1.5 mg.mL^−1^. The crystals were directly flash-frozen without any cryoprotectant. Data were collected at the ESRF (beamline ID30A-3) and processed with the XDS package^66^. The structure was solved by molecular replacement using the Influenza A virus R416A monomeric nucleoprotein structure (PDB ID code 3ZDP) without the oligomerization loop (from residues 391 to 439) as a model. Model building and refinement were performed using CCP4i suite program for crystallography (PHASER, ARP/wARP, REFMAC5, COOT)^67–71^. The final refinement was done using BUSTER^72^. The coordinates have been deposited in the Protein Data Bank under PDB ID code 5N2U. The protein structure figures were drawn using PyMOL^73^.

### Thermal shift assays

Thermal shift assays were performed following the established protocols of thermofluor experiments^74^ using a fluorescent probe (SYPRO Orange). SYPRO Orange dye binds non-specifically to hydrophobic surfaces and water strongly quenches its fluorescence. When a protein unfolds with the increase of the temperature, the exposed hydrophobic surfaces bind the dye, resulting in an increase of the fluorescence by excluding water. Sample were diluted at 0.25 mg.mL^−1^ in 50 mM Tris-HCl pH 7.5, 300 mM NaCl and 5 mM β-ME and 5X SYPRO Orange dye (Invitrogen) in a final volume of 40 µL. The thermal stability was measured using a real time PCR machine (Mx3005P Q-PCR, Stratagene). The dye was excited at 488 nm and the emission light was recorded at 585 nm while the temperature was increased by increments of 1 °C per minute from 25 to 75 °C. The relative fluorescence emission was then plotted against its corresponding temperature to produce the thermal shift profile curve. The melting temperatures were estimated from the derivative curves.

### Circular dichroism

Circular dichroism (CD) spectroscopy is an efficient tool for rapid determination of the secondary structure and folding properties of a protein by measuring the absorption difference between left and right circularly polarized light. A JASCO J-810 CD spectropolarimeter equipped with a temperature-controller (Peltier system) was used to record the far-UV CD spectrum of D/NP_TAIL_ at 6.4 μM in presence or in absence of trimethylamine N-oxide (TMAO). TMAO is a stabilizing chemical agent known to promote protein folding. It can be used with CD spectroscopy to assess putative folding of unfolded peptides and proteins. In addition to D/NP_TAIL_ without TMAO, final concentrations of 0.5, 1, 2 and 3 M TMAO were used in phosphate buffer (10 mM, pH 7.2) to validate the unfolded nature of D/NP_TAIL_. Due to the high absorbance of TMAO around 200 nm, spectra were recorded up to the point before the HT rose above 700 V (190 nm without TMAO, 205 nm with 0.5 M TMAO, 207 nm with 1 M TMAO, 210 nm with 2 M TMAO and 212 nm with 3 M TMAO). Spectra were collected at 20 °C with fifteen runs using a cuvette with a path-length of 1 mm. After blank subtraction, the CD signal (in mdeg) was converted to mean molar residue ellipticity (in deg.cm^2^.dmol^−1^).

### Surface plasmon resonance (SPR) measurements and analysis

The D/NP_TAIL_ sequence was fused to the sequence encoding a biotinylation motif (NGSGGGLNDIFEA-QKIEWHE) and cloned in pETM11 (EMBL). *E*. *coli* BL21 RIL (DE3) were transformed and grown up to an OD of 0.6–0.8 in a medium supplemented with biotin (12.5 μg. mL^−1^) in order to biotinylate the peptide *in vivo* during the expression (12 h, 18 °C, 0.3 mM IPTG). Purification was performed at room temperature in two steps, a Nickel affinity chromatography, followed by TEV cleavage and size exclusion chromatography (S75 10/300 GL, GE-Healthcare). A control biotinylated peptide (LEEMK-KGHLERECMEETCSYEEAREVFEDSEKTNEFWNK-biotin) was also used as negative control.

SPR experiments were carried out on a Biacore 3000 (GE Healthcare). Streptavidin was first immobilized on a CM5 sensor chip (GE Healthcare) surface through amine coupling at a flow rate of 5 μL.min^−1^. The biotinylated NP_TAIL_ (10 μg.mL^−1^) or the control biotinylated peptide (10 μg.mL^−1^) were diluted in surfactant-supplemented HBS-N running buffer (10 mM Hepes pH 7.4, 150 mM NaCl, 0.05% Tween 20) (GE Healthcare) and the attachment was carried out through biotin:streptavidin interaction, at a flow rate of 5 μL.min^−1^ up to 400–500 RU. A flow-cell containing immobilized streptavidin only was used as reference while the flow-cell with the streptavidin:biotinylated NP_TAIL_ complex was used as active flow-cell. For kinetic measurements, the importin-α7 analyte was serial-diluted in surfactant-supplemented HBS-N running buffer to final concentrations of 10, 25, 50, 75 and 100 nM. Each concentration was injected in triplicate in both the reference and active flow-cells. Analyte injection and following buffer injection times were set at 180 s and 150 s respectively at a flow-rate of 15 μL.min^−1^. As the analyte spontaneously and completely dissociated, no regeneration was required and complete dissociation was achieved in buffer for 300 s. All sensorgrams were reference-subtracted and injection points were aligned to be processed with the analysis. Kinetic data were analysed using the Biacore 3000 Evaluation software (GE Healthcare) under a Langmuir 1:1 binding model with mass-transfer. Chi^2^ values for the sensorgram fits used to determine the kinetic parameters were kept below 3, due to the obtained high signal, and T-value for rate parameters above at least 30. The residuals for the fitting were kept between 1 and −1.

### Nuclear import of WT and NP mutants in HEK 293T cells

#### Plasmids

The D/NP, D/NP-511 and D/NP-529 genes were amplified by PCR, cloned into the multiple cloning site of pSC-A-amp/kan vector (Strataclone blunt kit, Agilent) and expressed into competent bacterial cells, according to the manufacturer’s instructions. The NP genes in pSC-A-amp/kan vector were cloned into KpnI and BamHI restriction sites of an eukaryotic expression vector, pCDNA3.1 (+), driven by a CMV promoter. The constructs sequences were confirmed by Sanger sequencing (GATC Biotech platform, Germany).

#### Rabbit antibodies

To generate rabbit hyperimmune serum anti-D/NP, three 7 to 8 weeks old New Zealand white rabbits (Pôle Experimental Cunicole de Toulouse, Castanet-Tolosan) were immunized at day 0 and day 35 with 100–200 µg D/NP in incomplete Freund adjuvant. Rabbits were anesthetized and bled two weeks post boost (50 mL blood collected intra-cardiac before humane euthanasia).

#### Infection

Human embryonic kidney cells (HEK 293T; ATCC) were infected with D/bovine /France/5920/2014 at a multiplicity of infection of 5. Briefly, cells were washed with PBS, the inoculum was added and the cells were incubated 1 hour at 37 °C + 5% CO_2_. After incubation, infection media (OPTI-MEM) was added and the cells were incubated until the fixation.

#### Transfection

HEK 293T cells were grown on glass coverslips in Dulbecco’s modified Eagle’s medium (DMEM) complemented with 10% fetal bovine serum (FBS) and penicillin-streptomycin (Dominique DUTSCHER SAS) and incubated at 37 °C + 5% CO_2_ for 24 hours. Two hours before transfection, DMEM media was removed and replaced by OPTI-MEM media (Dominique DUTSCHER SAS). Cells were transfected with 2.5 µg of the respective plasmids using Mirus-TransIT reagent (Mirus) following the manufacturers’ instructions. Cells were then incubated for 24 h at 37 °C + 5% CO_2_.

#### Immunofluorescence

Cells were pre-fixed using 4% paraformaldehyde (Bio-Rad) for 10 minutes at room temperature and then, fixed and permeabilized using ice cold ethanol:acetone for 10 minutes at room temperature. After a blocking step using PBS complemented with 5% horse serum, cells were incubated with a rabbit hyperimmune D/NP serum at a 1:100 dilution for 2 hours at room temperature. After three washing steps with PBS-triton 0.05%, cells were incubated with anti-rabbit IgG labeled Rhodamine RX (Jackson Immuno; Research, 711.296.152) at 1:200 dilution for 1 hour in the dark and at room temperature. Then, cells were washed with PBS-triton 0.05% and coverslips were mounted on glass slides using Vectashield-DAPI mounting medium (Vector Laboratories) and analyzed with a Leica Zeiss 710 at the Cell Imagery Platform in Purpan (Toulouse).

### Ethics Statement

Experimentations were conducted in accordance with European and French legislations on Laboratory Animal Care and Use (French Decree 2001-464 and European Directive CEE86/609) and the animal protocol was approved by the Ethics Committee “Sciences et santé animale” number 115.

## Supplementary information

Supplementary information
