# Supplementary material for: The structure of the nucleoprotein of Influenza D shows that all Orthomyxoviridae nucleoproteins have a similar NPCORE, with or without a NPTAIL for nuclear transport
Source: Sci Rep. 2019 Jan 24;9:600. doi: 10.1038/s41598-018-37306-y (PMC6346101; doi:10.1038/s41598-018-37306-y)

The structure of the nucleoprotein of Influenza D shows that all *Orthomyxoviridae* nucleoproteins have a similar NP_{CORE}, with or without a NP_{TAIL} for nuclear transport

Amélie DONCHET[1], Justine OLIVA[2], Alice LABARONNE[1], Laura TENGO[1], Myriam MILOUDI[1], Francine C. A. GERARD[1], Caroline MAS[3], Guy SCHOEHN[1], Rob W. H. RUIGROK[1], Mariette DUCATEZ[2] and Thibaut CREPIN[1]*

[1] Institut de Biologie Structurale (IBS), Univ. Grenoble Alpes, CEA, CNRS, 38044 Grenoble, France

[2] IHAP, Université de Toulouse, INRA, ENVT, Toulouse, France

[3] Integrated Structural Biology Grenoble (ISBG) - UMS 3518 (CNRS-CEA-UJF-EMBL), 38044 Grenoble, France

Supplementary information

Supplementary Figure 1

Sequence alignment of NP from representative Influenza strains. The sequences used are the same as in Table 1. The figure has been drawn using ESPript [1]. The upper and lower secondary structures correspond to the X-ray structures of D/NP (PDB entry 5N2U) and A/NP (PDB entry 2IQH), respectively. The stars indicate the phosphorylation sites identified for A/NP (closed symbol) and B/NP (open symbol) by Hutchinson *et al.* (red)[2], Li *et al.* (green)[3] and Monda *et al.* (blue)[4].

Supplementary Figure 2

Effect of trimethylamine N-oxide (TMAO) on the folding of D/NP$_{TAIL}$. Far UV-circular dichroism spectra of either D/NP$_{TAIL}$ alone (purple) or in presence of TMAO (0.5 M in yellow, 1M in red, 2M in blue and 3 M in green) were collected. Because of the high background induced by the presence of TMAO around 200 nm, spectra were cut when the voltage (right bottom insert) became too high (> 700 V).

References

1 Robert, X. & Gouet, P. Deciphering key features in protein structures with the new ENDscript server. *Nucleic Acids Res* **42**, W320-324, doi:10.1093/nar/gku316 (2014).

2 Hutchinson, E. C. *et al.* Mapping the phosphoproteome of influenza A and B viruses by mass spectrometry. *PLoS Pathog* **8**, e1002993, doi:10.1371/journal.ppat.1002993 (2012).

3 Li, Y. *et al.* Phosphorylation and dephosphorylation of threonine 188 in nucleoprotein is crucial for the replication of influenza A virus. *Virology* **520**, 30-38, doi:10.1016/j.virol.2018.05.002 (2018).

4 Mondal, A. *et al.* Influenza virus recruits host protein kinase C to control assembly and activity of its replication machinery. *Elife* **6**, doi:10.7554/eLife.26910 (2017).

5 Chen, V. B. *et al.* MolProbity: all-atom structure validation for macromolecular crystallography. *Acta Crystallogr D Biol Crystallogr* **66**, 12-21, doi:10.1107/S0907444909042073 (2010).

Supplementary Table 1: Crystallographic data collection and structure refinement statistics. Values in parentheses are for highest resolution shell.

Crystal Parameters	D/NP
**Data collection**	
Beamline	ID30A3 - ESRF
Wavelength (Å)	0.9677
Space group	P1
Cell Dimensions a, b, c (Å)	75.17, 85.17, 103.39
α, β, γ (°)	91.20, 101.94, 101.02
Molecules per asymmetric unit	4
Wilson B-factor ($Å^2$)	51.4
Resolution (Å)	40 - 2.4 (2.48 - 2.4)
Observed Reflections	191 888 (18 695)
Unique Reflections	87 824 (8 505)
Completeness (%)	91.3 (94.1)
R_{sym} (I)[(a)] (%)	9.3 (84.7)
<I/σ(I)>	7.78 (1.05)
**Model quality indicators**	
R_{cryst} (%)	20.2 (21.9)
R_{free} (%)	25.2 (23.8)
Non-hydrogen atoms	14 646
Water atoms	235
Average B-factors ($Å^2$)	55.7
B-factors per chains ($Å^2$)	59.8, 54.3, 52.8, 56.3
B-factors water ($Å^2$)	49.5
Ramachandran favoured / allowed (%)	94.0 / 5.1
rms deviations, bonds (Å) / angles (°)	0.009 / 1.15
Clashscore[(b)]	4.31
MolProbity score[(b)]	2.28

[(a)] $R_{sym} = \sum_{hkl}\sum_j |I_{hkl,j} - \langle I_{hkl}\rangle| / \sum_{hkl}\sum_j I_{hkl,j}$

[(b)] calculated using MolProbity [5]

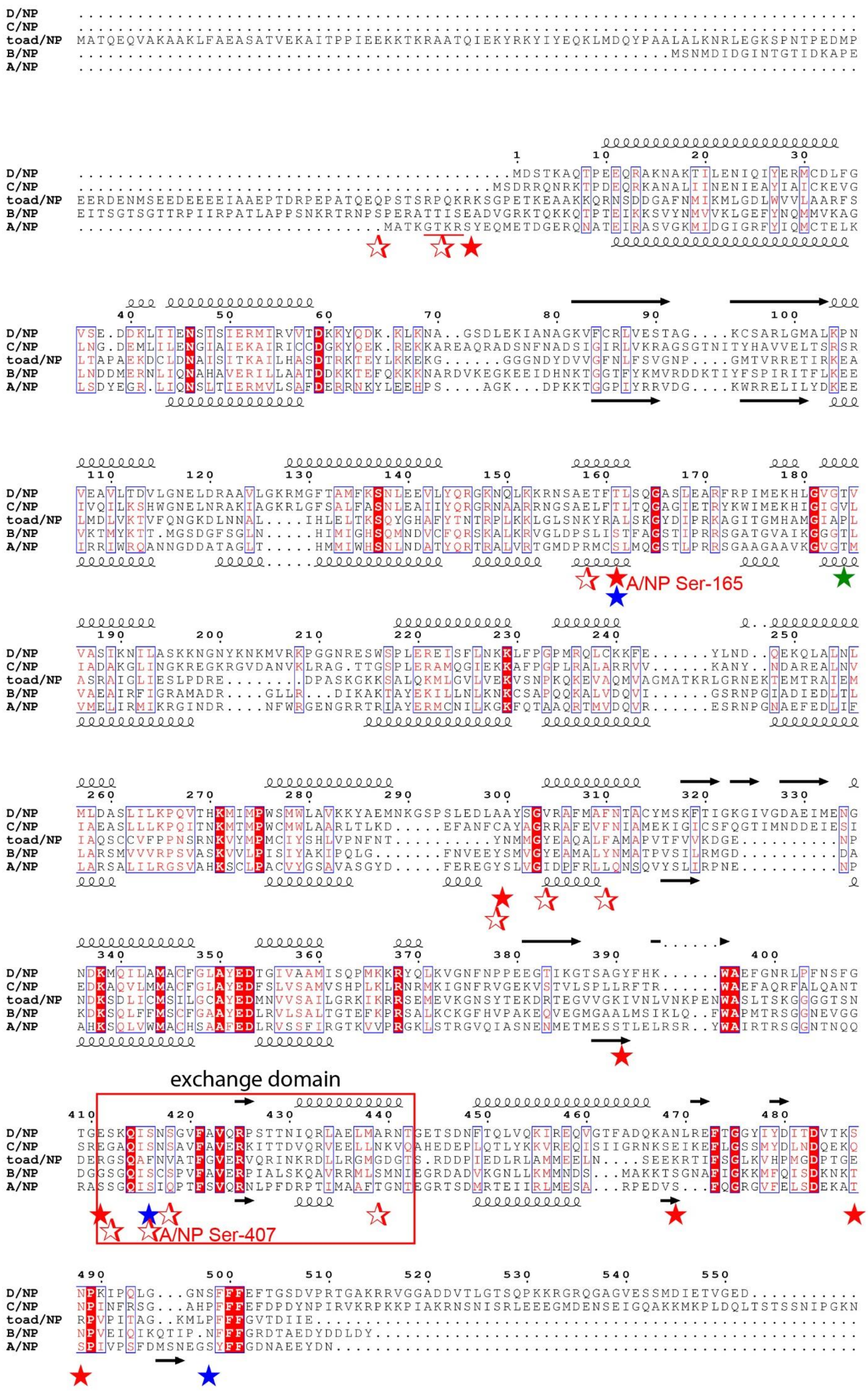
D/NP ....
C/NP ....
toad/NP MATQEQVAKAAKLFAEASATVEKAITPPIEEKKTKRAATQIEKYRKYIYEQKLMDQYPAALALKNRLEGKSPNTPEDMP
B/NP ....MSNMDIDGINTGTIDKAPE
A/NP ....
1 10 20 30
D/NP ....MDSTKAQTPEEQRAKNAKTILENIQIYERMCDLFG
C/NP ....MSDRRQNRKTPDEQRKANALIINENIEAYIAICKEVG
toad/NP EERDENMSEEDEEEIAAEPTDRPEPATQEQPSTSRPQKRKSGPETKEAAKKQRNSDDGAFNMIKMLGDLWVVLAARFS
B/NP EITSGTSGTTRPIIRPATLAPPSNKRTRNPSPERATTISEADVGRKTQKKQTPTEIKKSVYNMVVKLGEFYNQMMVKAG
A/NP ................................MATKGTKRSYEQMETDGERQNATEIRASVGKMIDGIGRFYIQMCTELK
40 50 60 70 80 90 100
D/NP VSE.DDKLIIENSISIERMIRVVTDKKYQDK.KLKNA..GSDLEKIANAGKVFCRLVESTAG....KCSARLGMALKPN
C/NP LNG.DEMLILENGIAIEKAIRICCDGKYQEK.REKKAREAQRADSNFNADSIGIRLVKRAGSGTNITYHAVVELTSRSR
toad/NP LTAPAEKDCLDNAISITKAILHASDTRKTEYLKKEKG......GGGNDYDVVGFNLFSVGNP....GMTVRRETIRKEA
B/NP LNDDMERNLIQNAHAVERILLAATDDKKTEFQKKKNARDVKEGKEEIDHNKTGGTFYKMVRDDKTIYFSPIRITFLKEE
A/NP LSDYEGR.LIQNSLTIERMVLSAFDERRNKYLEEHPS....AGK...DPKKTGGPIYRRVDG....KWRRELILYDKEE
110 120 130 140 150 160 170 180
D/NP VEAVLTDVLGNELDRAAVLGKRMGFTAMFKSNLEEVLYQRGKNQLKKRNSAETFTLSQGASLEARFRPIMEKHLGVGTV
C/NP IVQILKSHWGNELNRAKIAGKRLGFSALFASNLEAIIYQRGRNAARRNGSAELFTLTQGAGIETRYKWIMEKHIGIGVL
toad/NP LMDLVKTVFQNGKDLNNAL....IHLELTKSQYGHAFYTNTRPLKKLGLSNKYRALSKGYDIPRKAGITGMHAMGIAPL
B/NP VKTMYKTT.MGSDGFSGLN.....HIMIGHSQMNDVCFQRSKALKRVGLDPSLISTFAGSTIPRRSGATGVAIKGGGTL
A/NP IRRIWRQANNGDDATAGLT.....HMMIWHSNLNDATYQRTRALVRTGMDPRMCSLMQGSTLPRRSGAAGAAVKGVGTM
A/NP Ser-165
190 200 210 220 230 240 250
D/NP VASIKNILASKKNGNYKNKMVRKPGGNRESWSPLEREISFLNKKLFPGPMRQLCKKFE......YLND..QEKQLALNL
C/NP IADAKGLINGKREGKRGVDANVKLRAG.TTGSPLERAMQGIEKKAFPGPLRALARRVV......KANY..NDAREALNV
toad/NP ASRAIGLIESLPDRE........DPASKCKKSALQKMLQVLVEKVSNPKQKEVAQMVAGMATKRLGRNEKTEMTRAIEM
B/NP VAEAIRFIGRAMADR....GLLR...DIKAKTAYEKILLNLKNKCSAPQQKALVDQVI......GSRNPGIADIEDLTL
A/NP VMELIRMIKRGINDR....NFWRGENGRRTRIAYERMCNILKGKFQTAAQRTMVDQVR......ESRNPGNAEFEDLIF
260 270 280 290 300 310 320 330
D/NP MLDASLILKPQVTHKMIMPWSMWLAVKKYAEMNKGSPSLEDLAAYSGVRAFMAFNTACYMSKFTIGKGIVGDAEIMENG
C/NP IAEASLLLKPQITNKMTMPWCMWLAARLTLKD.....EFANFCAYAGRRAFEVFNIAMEKIGICSFQGTIMNDDEIESI
toad/NP IAQSCCVFPPNSRNKVYMPMCIYSHLVPNFNT.........YNMMGYEAQALFAMAPVTFVVKDGE..........NP
B/NP LARSMVVVRPSVASKVVLPISIYAKIPQLG........FNVEEYSMVGYEAMALYNMATPVSILRMGD..........DA
A/NP LARSALILRGSVAHKSCLPACVYGSAVASGYD.....FEREGYSLVGIDPFRLLQNSQVYSLIRPNE..........NP
340 350 360 370 380 390 400
D/NP NDKMQILAMACFGLAYEDTGIVAAMISQPMKKRYQLKVGNFNPPEEGTIKGTSAGYFHK......WAEFGNRLPFNSFG
C/NP EDKAQVLMMACFGLAYEDFSLVSAMVSHPLKLRNRMKIGNFRVGEKVSTVLSPLLRFTR......WAEFAQRFALQANT
toad/NP NDKSDLICMSILGCAYEDMNVVSAILGRKIKRRSEMEVKGNSYTEKDRTEGVVGKIVNLVNKPENWASLTSKGGGGTSN
B/NP KDKSQLFFMSCFGAAYEDLRVLSALTGTEFKPRSALKCKGFHVPAKEQVEGMGAALMSIKLQ..FWAPMTRSGGNEVGG
A/NP AHKSQLVWMACHSAAFEDLRVSSFIRGTKVVPRGKLSTRGVQIASNENMETMESSTLELRSR..YWAIRTRSGGNTNQQ
exchange domain
410 420 430 440 450 460 470 480
D/NP TGESKQISNSGVFAVQRPSTTNIQRLAELMARNTGETSDNFTQLVQKIREQVGTFADQKANLREFTGGYIYDITDVTKS
C/NP SREGAQISNSAVFAVERKITTDVQRVEELLNKVQAHEDEPLQTLYKKVREQISIIGRNKSEIKEFLGSSMYDLNDQEKQ
toad/NP DERGSQAFNVATFGVERVQRINKRDLLRGMGDGTS.RDDPIEDLRLAMMEELN....SEEKRTIFSGLKVHPMGDPTGE
B/NP DGGSGQISCSPVFAVERPIALSKQAVRRMLSMNIEGRDADVKGNLLKMMNDS...MAKKTSGNAFIGKKMFQISDKNKT
A/NP RASSGQISIQPTFSVQRNLPFDRPTIMAAFTGNTEGRTSDMRTEIIRLMESA...RPEDVS...FQGRGVFELSDEKAT
A/NP Ser-407
490 500 510 520 530 540 550
D/NP NPKIPQLG...GNSFFFEFTGSDVPRTGAKRRVGGADDVTLGTSQPKKRGRQGAGVESSMDIETVGED..........
C/NP NPINFRSG...AHPFFFEFDPDYNPIRVKRPKKPIAKRNSNISRLEEEGMDENSEIGQAKKMKPLDQLTSTSSNIPGKN
toad/NP RPVPITAG..KMLPFFFGVTDIIE..
B/NP NPVEIQIKQTIP.NFFFGRDTAEDYDDLDY..
A/NP SPIVPSFDMSNEGSYFFGDNAEEYDN..

Supplementary Figure 2

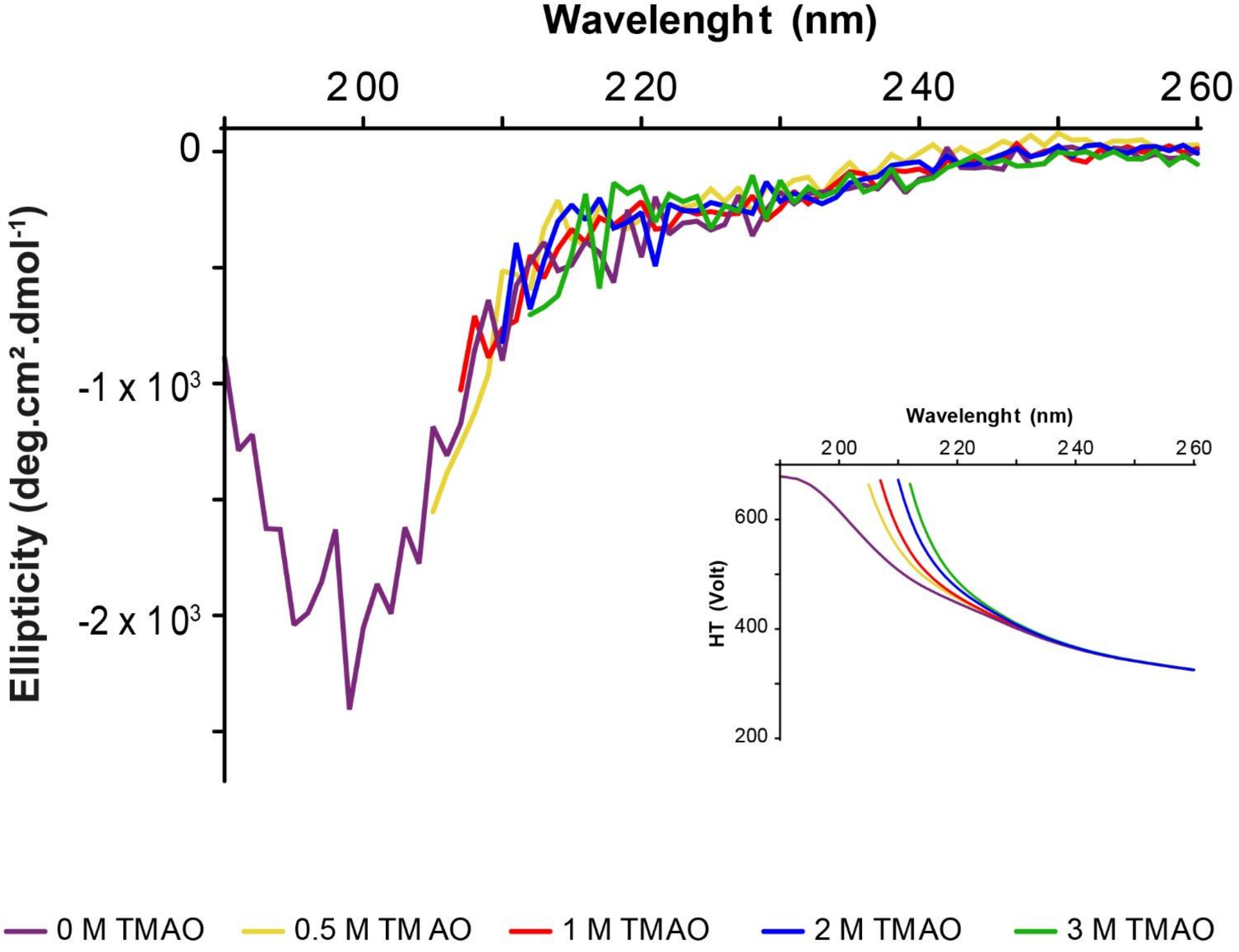

Supplement: Supplementary file 1 — Supplementary information [file 41598_2018_37306_MOESM1_ESM.pdf]
